# Structural and Functional Hippocampal Changes in Subjective Cognitive Decline From the Community

**DOI:** 10.3389/fnagi.2020.00064

**Published:** 2020-03-17

**Authors:** Lingyan Liang, Lihua Zhao, Yichen Wei, Wei Mai, Gaoxiong Duan, Jiahui Su, Xiucheng Nong, Bihan Yu, Chong Li, Xiaping Mo, Georgia Wilson, Demao Deng, Jian Kong

**Affiliations:** ^1^Department of Radiology, First Affiliated Hospital, Guangxi University of Chinese Medicine, Nanning, China; ^2^Department of Acupuncture, First Affiliated Hospital, Guangxi University of Chinese Medicine, Nanning, China; ^3^Department of Psychiatry, Massachusetts General Hospital, Harvard Medical School, Charlestown, MA, United States

**Keywords:** subjective cognitive decline, resting-state functional MR imaging, voxel-based morphometry, functional connectivity, hippocampus

## Abstract

**Background:**

Recently, subjective cognitive decline (SCD) has been described as the earliest at-risk state of Alzheimer’s disease (AD), and drawn attention of investigators. Studies suggested that SCD-community individuals may constitute a more vulnerable population than SCD-clinic patients, therefore, to investigate the early changes of the brain may provide guidance for treatment of the disease. We sought to investigate the changes of structure and functional connectivity alternation of the hippocampus in individuals with SCD recruited from the community using structural and resting-state functional MRI (fMRI).

**Methods:**

Thirty-five SCD patients and 32 healthy controls were recruited. Resting-state fMRI data and high-resolution T1-weighted images were collected. Whole-brain voxel-based morphometry was used to examine the brain structural changes. We also used the hippocampal tail and the whole hippocampus as seeds to investigate functional connectivity alternation in SCD.

**Results:**

Individuals with SCD showed significant gray matter volume decreases in the bilateral hippocampal tails and enlargement of the bilateral paracentral lobules. We also found that individuals with SCD showed decreased hippocampal tail resting-state functional connectivity (rsFC) with the right medial prefrontal cortex (mPFC) and the left temporoparietal junction (TPJ), and decreased whole hippocampus rsFC with the bilateral mPFC and TPJ. These brain region and FC showing significant differences also showed significantly correlation with Montreal Cognitive Assessment (MoCA) scores.

**Conclusion:**

Individuals with SCD recruited from the community is associated with structural and functional changes of the hippocampus, and these changes may serve as potential biomarkers of SCD.

**Clinical Trial Registration:**

The Declaration of Helsinki, and the study was registered in http://www.chictr.org.cn. The Clinical Trial Registration Number was ChiCTR-IPR-16009144.

## Introduction

Alzheimer’s disease (AD), the most common form of dementia, is increasing all over the world. According to the World Alzheimer Report (Patterson, 2018), there were about 50 million people worldwide living with dementia in 2018, and this number is expected to more than triple by 2050. However, there are no effective treatments for AD, and recent drugs targeting tau pathology and amyloid-β at the mild or moderate dementia stage have failed to treat the disease (Doody et al., 2014; Salloway et al., 2014). Moreover, the difficulties of treating mild cognitive impairment (MCI) suggests that treatment should be applied at an earlier stage of the disease (Sperling et al., 2011; Cheng et al., 2017).

Subjective cognitive decline (SCD) has been described as the earliest at-risk state of AD and increases the risk for developing MCI and future AD (Jessen et al., 2014; Hu et al., 2017). It refers to individuals who have self-perceived persistent decline in cognition while neuropsychological tests remain within the normal range (Jessen et al., 2014). Recently, SCD has been receiving increased attention as a risk factor for the development of AD (Jessen et al., 2014).

In addition, studies have suggested that the recruitment sources for SCD studies may have a significant influence on study outcomes. For instance Kuhn et al. (2019) found higher atrophy progression over time and a relatively smaller proportion of APOE ε4 carriers in patients from an SCD clinic compared SCD patients from the community. This finding reinforced their previous study, which found that those who seek consultation are at a higher risk of developing Alzheimer’s clinical syndrome (Perrotin et al., 2017). It indicated that SCD-clinic patients may constitute a more vulnerable population than SCD-community patients. They may be at increased risk for cognitive decline and possibly Alzheimer’s clinical syndrome (Kuhn et al., 2019).

In recent years, brain imaging has been used to investigate the structural and functional alterations of individuals with SCD. A number of papers on SCD have revealved reduced volume in medial temporal lobe (MTL) structures, including the hippocampus and entorhinal cortex (Jessen, 2006; Striepens et al., 2010; Meiberth et al., 2015; Perrotin et al., 2015). These brain alterations are similar to those found in MCI patients, suggesting that SCD may be a pre-MCI stage (Ling et al., 2018). Functional changes have also been detected in individuals with SCD. For example, task-based fMRI studies (Rodda et al., 2010; Hu et al., 2017) have identified neural network disruptions during cognitive processes in SCD compared with controls.

To our knowledge, few studies have been applied on SCD-community individuals. In this study, we combined voxel-based morphometry (VBM) analysis and resting-state functional connectivity to investigate the patterns of structural and functional brain alterations in individuals with SCD recruited from the community compared with healthy controls. We hypothesized that functional connectivity disruption with or without hippocampal atrophy may be detected in SCD-community individuals compared with healthy controls (HC).

## Materials and Methods

All research procedures were conducted in accordance with the Declaration of Helsinki, and the study was registered in http://www.chictr.org.cn.

### Participants

Thirty-five SCD subjects and 32 healthy control subjects were recruited from local communities between January 2016 and January 2018. All participants went through standard clinical assessments, including interviews with medical history, neurological examinations, and a series of neuropsychological tests. The neuropsychological tests were conducted by two neurologists and included the Chinese version of the Mini-Mental State Examination (MMSE), the Beijing version of the Montreal Cognitive Assessment (MoCA), Clinical Dementia Rating (CDR) Scale, Activities of Daily Living (ADL) Scale, and Geriatric Depression Scale (GDS). In addition, there were six neuropsychological tests. Specifically, delayed recall and recognition of Auditory Verbal Learning Test (AVLT), Animal Fluency Test, the 30-item Boston Naming Test (BNT), and Part A and Part B of the Trail Making Test (TMT) were used to evaluate participants’ three cognitive domains: memory, language, and attention/executive function.

### Inclusion/Exclusion Criteria

Based on the definition of SCD by Jessen et al. (2014), inclusion criteria were as follows: (1) Aged 55–75 years old; (2) Self-reported cognitive decline; (3) Normal general cognitive examination scores: MoCA-A: primary school and below >19, secondary school >22, university >24; MMSE: illiterate >17, primary school >20, junior school and above >24 points; CDR: 0.

**FIGURE 1:**
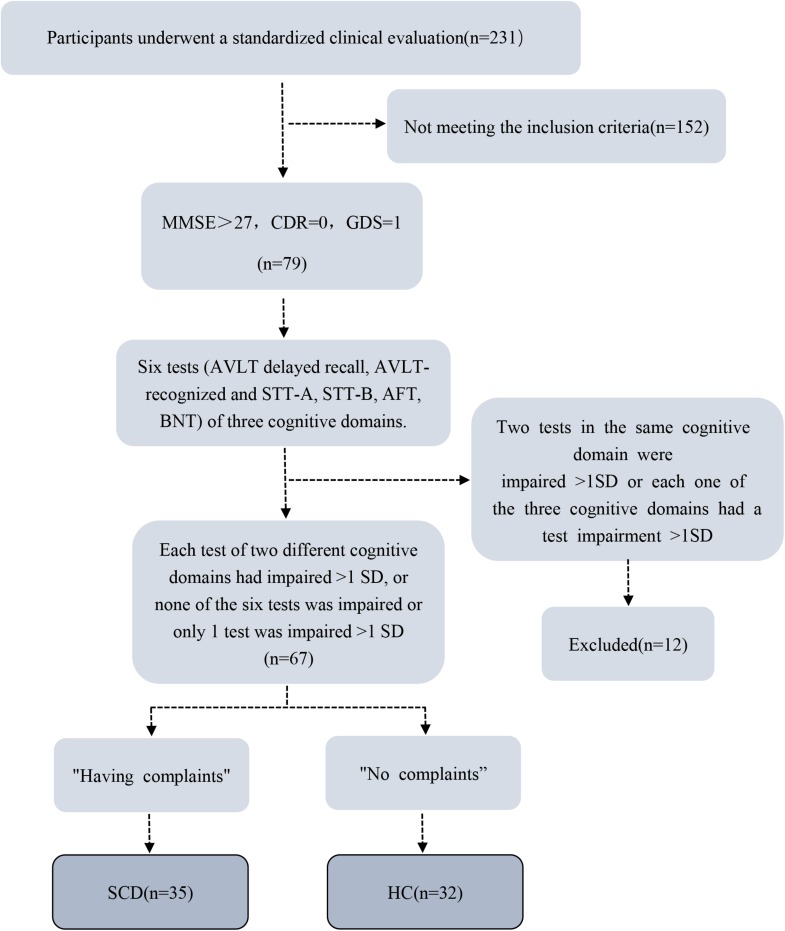
Flowchart shows details of data collection process. MMSE, Mini-Mental State Examination; CDR, Clinical Dementia Rating (CDR) Scale; GDS, Geriatric Depression Scale; AVLT, Auditory-Verbal Learning Test; STT, Shape Trails Test; AFT, Animal Fluency Test, BNT, Boston Naming Test; SCD, subjective cognitive decline; HC, healthy control; SD, standard deviation.

Exclusion criteria were: (1) MCI or dementia; (2) Vascular disease; (3) Severe depression; (4) Neurological diseases that may cause cognitive problems (such as brain tumors, Parkinson’s disease, encephalitis, epilepsy, etc.); (5) Brain trauma; (6) Other systemic diseases that can cause cognitive impairment, such as thyroid dysfunction, severe anemia, syphilis, HIV, etc.; (7) People with a history of mental illness or congenital intellectual disability; (8) Severe hearing or visual impairment, language communication disorders; (9) MRI contraindications (e.g., metal dentures or other metal implants that cannot be removed, claustrophobia, etc.); (10) Left-handed and double-handed (11) Non-handed elderly people. Details of the data collection process are exhibited as a flowchart (Figure 1).

### MRI Data Acquisition

All MRIs were performed with a 3.0T Siemens Magnetom Verio MRI System (Siemens Medical, Erlangen, Germany). To avoid head movement, each participant’s head was immobilized by foam pads in a standard 8-channel birdcage head coil. BOLD-fMRI images were obtained with a single-shot gradient-recalled echo planar imaging (EPI) sequence with the following parameters: repetition time (TR) / echo time (TE) = 2000 ms/30 ms, field of view (FOV) = 240 × 240 mm, flip angle = 90°, matrix size = 64 × 64, slice thickness = 5 mm, and slices = 31. High-resolution T1-weighted images were then acquired with a volumetric 3D spoiled gradient recall sequence with the following parameters: TR/TE = 1900 ms/2.22 ms, FOV = 250 mm × 250 mm, flip angle = 9°, matrix size: 256 × 256, slice thickness = 1 mm, and 176 slices.

### Brain Morphometry Data Analysis

The processing of structural MRI data was performed with the VBM12-toolbox^1^ incorporated in Statistical Parametric Mapping 12 (SPM12^2^). Each MRI image was first segmented into gray matter (GM), white matter (WM), and cerebrospinal fluid (CSF) using VBM12-toolbox. Then, the GM images were normalized to the Montreal Neurological Institute (MNI) space using the Diffeomorphic Anatomic Registration of the using Exponentiated Lie (DARTEL) algorithm. The registered gray matter partitions were multiplied by Jacobian determinants with only non-linear warping to exclude individual differences in total intracranial volume. Finally, modulated warped GM segments were resliced to an isotropic voxel-size of 1.5 mm^3^. Volumes were automatically determined from the modulated warped resliced GM segments. The GM segments were smoothed with a Gaussian smoothing kernel of 8 mm full-width at half maximum (FWHM).

Group analysis was applied using a two-sample *t*-test in SPM 12 with gender, age, GDS, years of education, and whole brain volume as covariates. A voxel-wise threshold of *p* < 0.001 (uncorrected) and a cluster-level threshold of *p* < 0.05 (family-wise error correction) were applied for data analysis. In addition, given the important role of the hippocampus and medial prefrontal cortex (mPFC) in memory processing (Tzourio-Mazoyer et al., 2002; Risacher et al., 2009, 2010; Verfaillie et al., 2016), we predefined the left / right hippocampus and MPFC as regions of interest and prepared templates using the WFU PickAtlas tool^3^. A voxel-wise threshold of *p* < 0.005 and a cluster-level threshold of *p* < 0.05 corrected using 3dClustSim were applied for the regions of interest data analysis.

### Resting State Functional Connectivity Data Analysis

Preprocessing was performed with CONN (CONN^4^). The first five volumes of each functional time series were removed to avoid the instability of the initial MRI signal. The remaining images were corrected for acquisition time delay between different slices and realigned to the first volume. The head motion parameters were calculated by estimating the translation in every direction and the angular rotation on each axis for every volume.

The realigned functional images were then spatially normalized to the Montreal Neurological Institute (MNI) space using the normalization parameters estimated by T1 structural image unified segmentation and re-sampled to 3 mm × 3 mm × 3 mm voxels. Several sources of spurious variance, such as the estimated motion parameters and average BOLD signals in ventricular and white matter regions, were removed from the images. After removing the variance, linear drift was removed and a temporal filter (0.01–0.08 Hz) was then performed on the time series of each voxel to reduce the effect of low-frequency drifts and high-frequency noise. Artifact detection toolbox was used to further correct head motion.

In this study, the regions of interest (seeds) were 1) hippocampus areas that showed significant difference between the SCD and controls derived from VBM analysis (bilateral hippocampal tail) and 2) bilateral whole hippocampus as defined by AAL atlas (Tzourio-Mazoyer et al., 2002).

The mean BOLD time course was extracted from the selected seeds of each subject. Pearson correlation coefficients were estimated between the mean time course of the seed region and the time courses of all other voxels. Pearson correlation coefficients were then normalized to z-scores with Fisher’s r-to-z transformation to acquire the entire brain z-score map of each subject for each condition.

A two-sample *t*-test was applied in SPM12 to compare the differences between the two groups. A voxel-wise threshold of *p* < 0.001 (uncorrected) and a cluster-level threshold of *p* < 0.05 (family-wise error corrected) were applied for data analysis. Age, gender, GDS, and years of education were included as covariates.

### Statistical Analysis

Demographic and clinical data were analyzed in SPSS 22.0 (SPSS, Inc.). Measured data were expressed as mean ± standard deviation. A two-sample *t*-test was used to compare age, years of education, and MoCA. Sex between groups was compared using a Pearson chi-squared test. Comparisons of clinical difference among the two groups (HC and SCD) were performed with an analysis of covariance (ANCOVA) and all demographic factors (gender, age, GDS, years of education, and whole brain volume) as covariates. Correlation analysis was used to estimate the relationship between MoCA and AVLT scores and neuroimaging findings in SCD and HC (*p* < 0.05 was considered significant).

## Results

### Demographic and Clinical Results

Thirty-five SCD and 32 HC subjects were included in our study. There were no significant differences in age, sex, education, and MoCA between SCD subjects and HC subjects (Table 1).

**TABLE 1:** Demographics and cognitive scores of healthy control and SCD groups.

**Demographics and scores**	**Control subjects (*n* = 32)**	**Patients with SCD (*n* = 35)**	***P*-value**
Age (y)	63.03 ± 5.433	64.94 ± 5.955	0.176
Sex			0.496
No. of men	12	16	
No. of women	20	19	
Education (y)	11.56 ± 2.994	12.29 ± 3.140	0.339
AVLT: delayed recall	6.22 ± 1.85	5.29 ± 1.71	0.035
AVLT: recognition	22.13 ± 1.60	22.03 ± 1.40	0.794
MMSE	29.13 ± 0.660	28.94 ± 0.838	0.330
MoCA	26.50 ± 1.832	26.16 ± 2.756	0.475
GDS	4.97 ± 2.307	5.26 ± 2.894	0.665

*Data are means ± standard deviation unless otherwise noted. The comparisons of demographic and cognitive scores between groups were performed with two-sample *t*-tests. *P* < 0.05 indicated a significant difference. AVLT, Auditory Verbal Learning Test; MMSE, Mini-Mental State Examination (Chinese Version); MoCA, Montreal Cognitive Assessment (Beijing Version); GDS, Geriatric Depression Scale (Chinese Version).*

### Brain Volume Analysis

**FIGURE 2:**
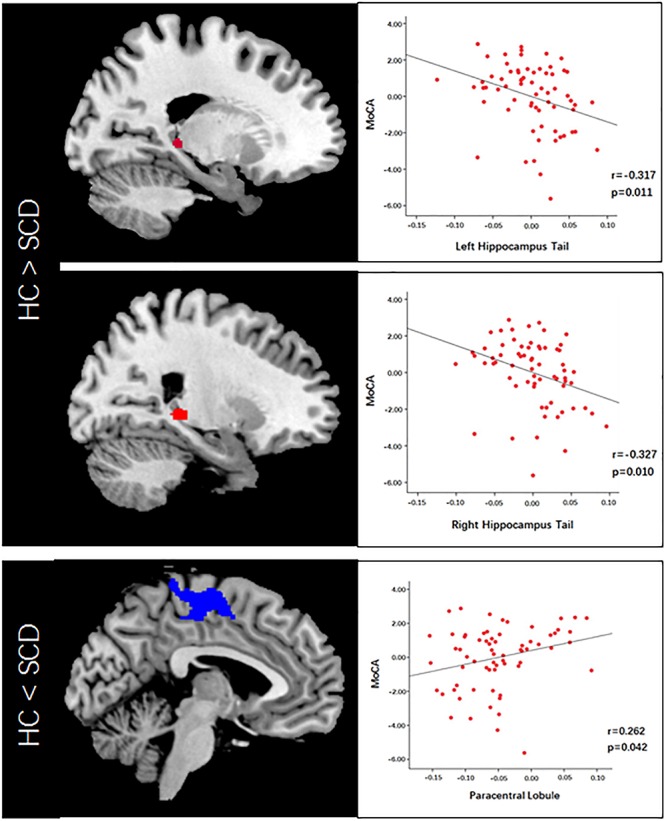
Voxel-based morphometry (VBM) analysis revealed altered gray matter volume in the SCD group compared to the HC group. Scatter plots illustrate MoCA scores and VBM correlation analysis.

VBM analysis showed that SCD is associated with significantly decreased gray matter volume (GMV) in the bilateral hippocampal tails and increased GMV in the bilateral paracentral lobules compared with HC subjects (Figure 2 and Table 2).

**TABLE 2:** Abnormal brain volume in SCD compared with HC.

**Brain region**	**Cluster size**	**Peak coordinates (mm)**			**Volume size**
		**X**	**Y**	**Z**	
Right HIP-t	100	23	−33	0	SCD<HC
Left HIP-t	52	−18	−35	2	SCD<HC
B-paracentral lobules	2373	−4	−16	60	SCD>HC

*HIP-t, hippocampal tail; B-paracentral lobules, bilateral paracentral lobules; SCD, subjective cognitive decline; HC, healthy control.*

**FIGURE 3:**
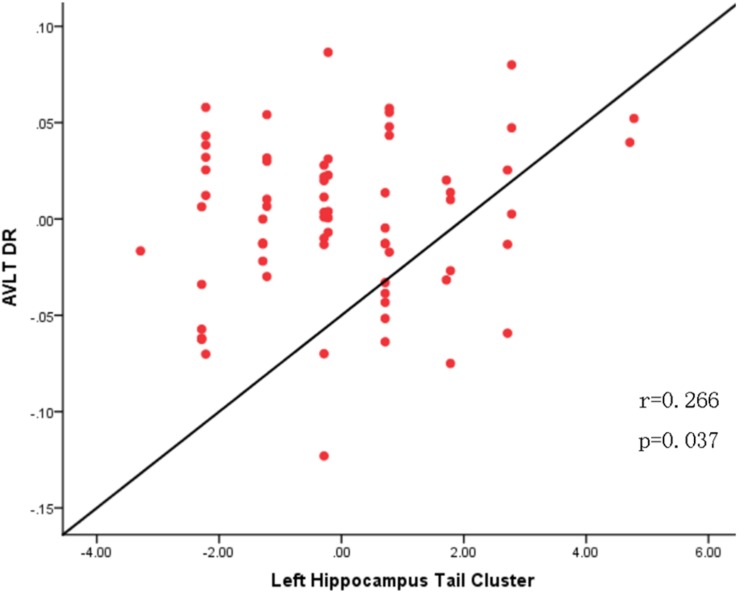
Correlation analysis between AVLT-Delayed recall scores and altered gray matter volume of the left hippocampus tail in the SCD group.

To explore the association between the VBM and clinical outcomes, we applied a correlation analysis between brain areas showing significant VBM difference and MoCA scores and found the decreased GMV of the left hippocampal tail was negatively correlated with MoCA scores[*r* = −0.317, *p* < 0.011, significant after Bonferroni correction (0.05/3)], the similar correlation was also found between the right hippocampal tail and MoCA scores (*r* = −0.327, *p* = 0.010, significant after Bonferroni correction). The increased GMV of the bilateral paracentral lobules showed a trend to positive correlation with MoCA scores. Exploratory (*r* = 0.262, *p* = 0.042, not significant after Bonferroni correction) across all subjects. Exploratory analysis showed that the decreased left hippocampal tail volume was significantly positive correlated with AVLT-delayed recall (dr) scores (*r* = 0.266, *p* = 0.037) (Figures 2, 3).

### Resting State Functional Connectivity Results

**FIGURE 4:**
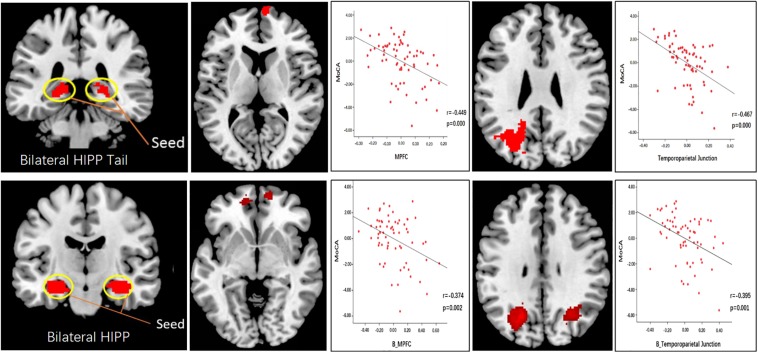
Functional connectivity analysis results using the hippocampal tail and whole hippocampus as seed. Scatter plots indicate the association between the MoCA scores and connectivity correlation analysis.

With the bilateral hippocampal tail as the seed, SCD subjects showed decreased rsFC with the right mPFC and the left temporoparietal junction (TPJ) compared to HC subjects (Figure 4 and Table 3). With the whole hippocampus as the seed, similar results were observed (i.e., SCD subjects showed decreased FC with the bilateral mPFC and the bilateral TPJ compared to HC subjects) (Figure 4 and Table 3).

**TABLE 3:** Abnormal functional connectivity in SCD compared with HC.

**Seed**	**Brain region**	**Cluster size**	***T*-value**	**Peak coordinates (mm)**			**Functional connectivity strength**
				**X**	**Y**	**Z**	
Bi HIP-t	Right mPFC	86	3.61	8	68	8	SCD<HC
	Left TPJ	591	3.99	−34	−66	26	SCD<HC
Bi whole HIP	Left mPFC	102	3.22	−12	52	−8	SCD<HC
	Right mPFC	151	3.65	16	54	−18	SCD<HC
	Left TPJ	1479	4.26	−22	−66	32	SCD<HC
	Right TPJ	1077	4.05	36	−56	50	SCD<HC
	Left mPFC	203	3.98	−18	34	−20	SCD<HC

*Bi, bilateral; HIP, hippocampus; HIP-t, hippocampal tail; mPFC, medial prefrontal cortex; TPJ, temporoparietal junction; SCD, subjective cognitive decline; HC, healthy control.*

To explore the association between functional connectivity and clinical outcomes, we applied a correlation analysis between brain areas showing significant functional connectivity difference and MoCA scores, and we found that MoCA scores were negatively correlated with the decreased bilateral hippocampus tails-right mPFC rsFC (*r* = −0.449, *p* < 0.001, significant after Bonferroni correction), bilateral hippocampus tail-left TPJ rsFC (*r* = −0.467, *p* < 0.001, significant after Bonferroni correction),bilateral hippocampus-bilateral mPFC rsFC (*r* = −0.374, *p* = 0.002, significant after Bonferroni correction) and bilateral hippocampus-bilateral TPJ rsFC (*r* = −0.395, *p* = 0.001, significant after Bonferroni correction) across all subjects (Figure 4).

## Discussion

In this study, we investigated the differences of brain morphometry and resting state functional connectivity (rsFC) between SCD (recruited from the community) and controls. We found that SCD is associated with a GMV decrease in the bilateral hippocampal tails and a GMV increase in the bilateral paracentral lobules. In addition, SCD subjects showed decreased hippocampus rsFC with the mPFC and TPJ.

Hippocampus plays an important role in memory processing and is considered among the first regions affected in AD pathological process (Bradley et al., 2002), of which the decreased volume is one of the most validated and widely used biomarkers of AD (de Flores et al., 2015). Converging evidences demonstrated total (Striepens et al., 2010) or partial (Cherbuin et al., 2015; Yue et al., 2018) hippocampus volume decreased in SCD. In the present study, we found decreased GMV of bilateral hippocampal tails in individuals with SCD which was consistent with previous studies. In addiction, the decreased left hippocampus tail GMV was significantly positive correlated with AVLT-dr scores which showed significant group difference. Studies revealed that AVLT is among the most sensitive episodic memory tests (Simard and van Reekum, 1999) and the delayed recall test was indicated to best identify early AD as well as predict the conversion to AD (Chen et al., 2000; Tierney et al., 2005). It is universally known that episodic memory is the first cognitive domain involved in the AD spectrum and the most sensitive neuropsychological indicator for early AD (Dubois et al., 2007). The AD pattern of gray matter was found in SCD which correlated with episodic memory decline (Peter et al., 2014), and RAVLT demonstrated significant association with the Hippocampal Occupancy Score and hippocampal volumes in the AD spectrum (Sudo et al., 2019). However, the change of hippocampus tends to be asymmetry in SCD, and the structural MRI studies revealed a decreased volume preferred the right hippocampus (Yue et al., 2018). While the verbal information may depend on the left hippocampus (Papanicolaou et al., 2002), which supports our result.

We also found an increased GMV in the paracentral lobule in individuals with SCD, which has not been previously reported. The paracentral lobule is comprised in the sensorimotor network and involved in the primary sensation and movement of the lower back, legs, and feet, as well as control of the bladder and bowel function. The alterations of pathology and structure in the sensorimotor cortices was least observed in aMCI and they are found to be preserved in AD relatively (Pearson et al., 1985; Frisoni et al., 2009). Accordingly, the increased ReHo found in the sensorimotor network might be interpreted as compensatory mechanism to the functional disruptions of other brain networks (Zhen et al., 2018). However, study with the result of functional abnormality in aMCI (Zhao et al., 2014) provided support to the assumption that part of the motor areas might have cognitive functions (Hanakawa et al., 2002). Decreased volume in the paracentral lobule was found in AD (Yi et al., 2009). A recent study revealed altered subnetworks including paracentral lobule which were correlated with the scores of the neurocognitive assessments in SCD (Kim et al., 2019). We speculate that the enlargement of the paracentral lobule may reflecting a potential compensatory mechanism. It is still uncertain whether it has underlying correlation with cognitive function or be affected indirectly by the disruption of other brain regions. Further studies are needed to validate our finding.

We identified significant disrupted connectivity between the hippocampal tail/whole hippocampus and the mPFC and TPJ, and these disruptions were negatively correlated with MoCA scores. These areas are all important regions of the default mode network (DMN) (Buckner et al., 2008), which has been vulnerable to AD. It is worth noting that using the whole hippocampus as a seed resulted in more extensive functional connectivity changes than using only the hippocampal tail as a seed. Studies suggest that hippocampal subregions may play different roles (Knierim et al., 2006; Zeidman and Maguire, 2016) and are affected differently in patients with AD (Arnold et al., 1991; Braak and Braak, 1991). Wang et al. (2006) and de Flores et al. (2017) used the hippocampal subfields or the whole hippocampus as seeds and revealed functional connectivity disruption in brain regions including the mPFC, posterior cingulate cortex, precuneus, temporal lobe, and angular cortex in AD and SCD. Our results are consistent with previous studies and provide indirect support for a previous hypothesis that functional abnormality may occur before structural alteration in patients with SCD (Sun et al., 2016).

Literature suggests that communication between the hippocampus and mPFC plays an important role in memory processes (van Kesteren et al., 2012; Kurczek et al., 2015; Tao et al., 2019). For instance, Van Kesteren et al. (van Kesteren et al., 2012, 2013) investigated how connections between the hippocampus and the mPFC relate to the incorporation of new memories into existing abstract frameworks. They found that hippocampus-mPFC connectivity is enhanced during and shortly after the successful encoding of novel information. Ries et al. (2012) found that functional connectivity between the hippocampal tail and mPFC was associated with accuracy of memory self-rating, which indicates that hippocampus-mPFC functional connectivity is closely related to memory. Investigators have also found decreased functional connectivity between the right hippocampus and superior mPFC in SCD (Hu et al., 2016). In a previous study, we found that mind-body intervention can significantly modulate hippocampus-mPFC rsFC and that this change is associated with memory function changes (Tao et al., 2016). Thus, our results align with previous findings.

The TPJ refers to an area of the cortex at the junction of the posterior superior temporal sulcus, inferior parietal lobule, and lateral occipital cortex. A brain imaging study (Donaldson et al., 2015) suggested that the TPJ is involved in various processes such as episodic memory retrieval, attention, language and speech, temporal processing, social cognition and resting state activity. Kucyi et al. (2012) investigated functional connectivity between the bilateral TPJ and other brain regions, such as the prefrontal, middle cingulate cortex, and insula, and found that the right TPJ functional connectivity was strongest with the ventral attention network, while left TPJ connectivity was strongest with the executive control network (ECN). Consequently, altered functional connectivity between the hippocampus and TPJ may contribute to the abnormal memory and attention function in SCD.

Montreal Cognitive Assessment is an efficient and rapid screening tool for cognitive dysfunction involving memory, executive function, and attention (Jiang et al., 2018) and has moderate specificity and high sensitivity (Mclennan et al., 2011). Previous studies have found that MoCA scores significantly correlate with both structural and functional brain alterations in SCD (Sun et al., 2016; Shu et al., 2017). We also observed a significant correlation between MoCA scores and both structural and functional alterations in the current study, which shed light on the significance of these brain function and morphometry findings.

There are several potential limitations to the present study. First, our results were limited by the relatively small sample sizes. Future studies with larger sample sizes are needed to further confirm our findings. Second, the participants involved in our study were only recruited from the community. SCD participants recruited from hospitals/clinics (i.e., medical help-seeking individuals) should be involved in future studies, and a comparison should be between these two groups. Third, we only included structural MRI and rs-fMRI. Future studies could incorporate relevant biomarkers such as β-amyloid and tau proteins. Finally, the present study was cross-sectional. A longitudinal study is needed to assess whether structural and functional alterations in SCD could predict the development of the disease.

In summary, we found that SCD individuals recruited from the community is associated with decreased GMV at the hippocampal tail, increased GMV at the paracentral lobules, and decreased hippocampus rsFC with the mPFC and TPJ. Our findings may shed new light on the neuroimaging biomarker of SCD.

## Data Availability Statement

The raw data supporting the conclusions of this article will be made available by the authors, without undue reservation, to any qualified researcher.

## Ethics Statement

The studies involving human participants were reviewed and approved by the Medicine Ethics Committee of the First Affiliated Hospital, Guangxi University of Chinese Medicine. The patients/participants provided their written informed consent to participate in this study. Written informed consent was obtained from the individual(s) for the publication of any potentially identifiable images or data included in this manuscript.

## Author Contributions

DD and JK provided the theory behind this work and designed the experiment. LL and LZ made substantial contributions to the present study and revised and handled the manuscript. GD was mainly responsible for image processing and statistical analysis. YW, CL, WM, JS, XN, and BY contributed to sample collection. JK and XM reviewed data and provided the critical comments or suggestions. GW had primary responsibility for final content.

## Conflict of Interest

The authors declare that the research was conducted in the absence of any commercial or financial relationships that could be construed as a potential conflict of interest.

## Footnotes

1 http://dbm.neuro.uni-jena.de/vbm/

2 http://www.fil.ion.ucl.ac.uk/spm/

3 https://www.nitrc.org/projects/wfu_pickatlas/

4 http://www.nitrc.org/projects/conn
